# MicroRNAs hsa-miR-618 and hsa-miR-297 Might Modulate the Pleiotropic Effects Exerted by Statins in Endothelial Cells Through the Inhibition of ROCK2 Kinase: *in-silico* Approach

**DOI:** 10.3389/fcvm.2021.704175

**Published:** 2021-08-16

**Authors:** Karla Leal, Kathleen Saavedra, Camilo Rebolledo, Luis A. Salazar

**Affiliations:** Center of Molecular Biology and Pharmacogenetics, Scientific and Technological Bioresource Nucleus, Department of Basic Sciences, Faculty of Medicine, Universidad de La Frontera, Temuco, Chile

**Keywords:** statins, pleiotropic effects, microRNAs, atheroprotective effects, kinases

## Abstract

Several studies show that statin therapy improves endothelial function by cholesterol-independent mechanisms called “pleiotropic effects.” These are due to the inhibition of the RhoA/ROCK kinase pathway, its inhibition being an attractive atheroprotective treatment. In addition, recent work has shown that microRNAs, posttranscriptional regulators of gene expression, can affect the response of statins and their efficacy. For this reason, the objective of this study was to identify by bioinformatic analysis possible new microRNAs that could modulate the pleiotropic effects exerted by statins through the inhibition of ROCK kinases. A bioinformatic study was performed in which the differential expression of miRNAs in endothelial cells was compared under two conditions: Control and treated with simvastatin at 10 μM for 24 h, using a microarray. Seven miRNAs were differentially expressed, three up and four down. Within the up group, the miRNAs hsa-miR-618 and hsa-miR-297 present as a predicted target to ROCK2 kinase. Also, functional and enriched pathway analysis showed an association with mechanisms associated with atheroprotective effects. This work shows an *in-silico* approach of how posttranscriptional regulation mediated by miRNAs could modulate the pleiotropic effects exerted by statins on endothelial cells, through the inhibition of ROCK2 kinase and its effects.

## Introduction

Cardiovascular diseases (CVDs) are the leading cause of death worldwide. An estimated 17.9 million people died from CVDs in 2016, representing 31% of all global deaths. Of these deaths, 85% are due to heart attack and ischemic stroke (1). Most CVDs can be prevented by addressing lifestyle-related risk factors: tobacco use, physical inactivity, hypertension, diabetes, and hyperlipidemia (2). The treatment of choice for hypercholesterolemia is the use of statins, which inhibit the enzyme 3-hydroxy-methylglutaryl coenzyme A (HMG-CoA) reductase, which limits the biosynthesis of liver cholesterol (3). Experimental and clinical studies suggest that statins may exert cardiovascular protective effects by mechanisms independent of cholesterol lowering called “pleiotropic effects” (4, 5). It has been described that the mechanism by which statins produce their pleiotropic effects is through inhibition of the mevalonate pathway. Mevalonate is a precursor not only of cholesterol but also of several other isopropenoids (6). These include farnesylpyrophosphate (FPP) and geranylgeranylpyrophosphate (GGP), which are lipids needed for posttranslational prenylation of Rho GTPase proteins, which act as biological switches through signal transduction (6, 7). The inhibition of mevalonate synthesis prevents the activation of Rho and the subsequent activation of Rho-associated protein kinase (ROCK) (8, 9). Several studies have reported the importance of the RhoA/ROCK pathway in endothelial function affecting vascular tone, platelet aggregation, smooth-muscle cell proliferation, leukocyte adhesion, production of nitric oxide synthase (eNOS), and bioavailability of nitric oxide (NO). The mechanisms associated with eNOS are of great importance, since the decrease of NO corresponds to one of the first manifestations of atherosclerosis (10, 11). Therefore, inhibition of the RhoA/ROCK pathway by statins influences NO signaling in endothelial cells (12). This has been demonstrated by two mechanisms: stability of eNOS messenger RNA (mRNA) and an increase in its activity through activation of phosphatidylinositol 3-kinase (PI3K)/AKT (13, 14). For this reason, inhibition of the RhoA/ROCK kinase pathway might be an attractive atheroprotective treatment for the development of CVD.

MicroRNAs (miRNAs) are important regulators of gene expression that bind complementary target mRNAs and repress their expression. The miRNAs are small non-coding RNA molecules, evolutionarily conserved. They have approximately 22 nucleotides, and their functions are gene regulation through posttranscriptional mechanisms. The miRNAs are involved in a variety of biological processes (15). Over the past few years, a total of 2,300 miRNAs have been reported in humans and it is estimated that human mRNA information exceeds 25,000 (16, 17). For this reason, current laboratory technologies do not allow to test globally every possible interaction between gene and miRNAs. However, the use of bioinformatics approaches for miRNA target prediction is used as a guide for laboratory validation experiments to more quickly elucidate gene regulation networks (18). The bioinformatics development combined with diverse experiments has allowed them to be used as potential biomarkers for diagnosis, prognosis, and personalized treatment (19).

Several studies in the area of cancer have demonstrated the regulation of transcriptional expression by miRNAs of the ROCK1 and ROCK2 isoforms of the RhoA/ROCK kinase pathway, reporting beneficial effects such as decreased proliferation and migration by modulating the expression of these miRNAs (20, 21). Zambrano et al. (22) showed that treatment with atorvastatin and simvastatin at 10 micromolar (μM) in HepG2 cells for 24 h deregulated the expression of 13 miRNAs by atorvastatin and 2 miRNAs by simvastatin. Other studies also show that atorvastatin can lower miR-221 and miR-222 levels in cell endothelial parents of patients with coronary disease (23). In particular, the effects observed by statins are heterogeneous, appearing to be independent of their chemical composition (lipophilic vs. hydrophilic) (24). It has also been reported that miRNAs would have the ability to influence endothelium atheroprotective effects, such as increasing nitric NO. Cerda et al. (25) described that these miRNAs increase the levels of NO and the expression of eNOS3 in endothelial cells. This background reveals that statin-modulated miRNAs influence their response and effectiveness. For this reason, the objective of this study was to identify by bioinformatic analysis possible new miRNAs that could modulate the pleiotropic effects exerted by statins through the inhibition of ROCK1 and ROCK2 kinases, and also to explore the pathways associated with the atheroprotective capacity of endothelial cells through an *in-silico* approach. Therefore, a bioinformatics study was performed comparing the differential expression of miRNAs in human umbilical vein endothelial cells (HUVEC) under two conditions: control and treated with simvastatin at 10 (μM) for 24 h.

## Materials and Methods

### Identification of Differential Expression miRNAs

The gene expression profile data (GSE126290, file GSE126290_RAW.tar) was downloaded from the NCBI Gene Expression Omnibus (GEO) database. The dataset contained the miRNA profile of HUVEC cells treated with simvastatin 10 μM and HUVEC cells without treatment as control. The analysis was performed using the Gene Expression Analysis Platform version 0.3.2 (GEAP), which uses R program packages for gene expression data processing and statistical analysis (26–30). In this analysis, the statistical package used was “Linear models and differential expression for microarray data” (Limma), making the comparison between groups considering as criteria the “false discovery rate” (FDR) *p*-value < 0.05 and logFC > 1 (31). From GEAP software, volcano plot, scatter plot, and heat map of the distances between arrays were generated. For principal component analysis (PCA), Ggfortify (32) and ggbiplot (33) of R Program were used. For the hierarchical clustering heat maps, Morpheus was used.

### Gene Ontology and Pathway Enrichment Analysis

To predict the target genes of the prognostic miRNAs, we used the mirDIP 4.1 online software that provides 152 million predictions using 30 different resources and obtained the score of each interaction between miRNAs and the target (34). In addition, gene ontology (GO) and pathway enrichment analyses of Kyoto Encyclopedia of Genes and Genomes (KEGG) were performed with target genes of the differential miRNAs expressed through the ShinyGO v0.61 (35) program, FDR *p*-value < 0.05.

### Target Predictions of Upregulated miRNAs for ROCK1 and ROCK2 Kinases

The upregulated miRNAs were evaluated for their ability to bind to the 3′UTR of the ROCK1 and/or ROCK2 kinases using three miRNA target predictor softwares to increase specificity and precision: TargetScan version 7.2, which considered as criteria the type of seed region and the weighted context++ score (WCS) that uses 16 important characteristics for miRNA target recognition (36); miRDB which considered as criteria the location of the seed region in the 3′UTR and the score assigned by the computational algorithm for target prediction, showing that a score above 80 presents a higher probability of union (37); and mirDIP 4.1.

## Results

### Identification of Differentially Expressed miRNAs Between Simvastatin-Treated 10 μM HUVEC Cells (24 h Treatment) and Control HUVEC Cells

**Figure 1:**
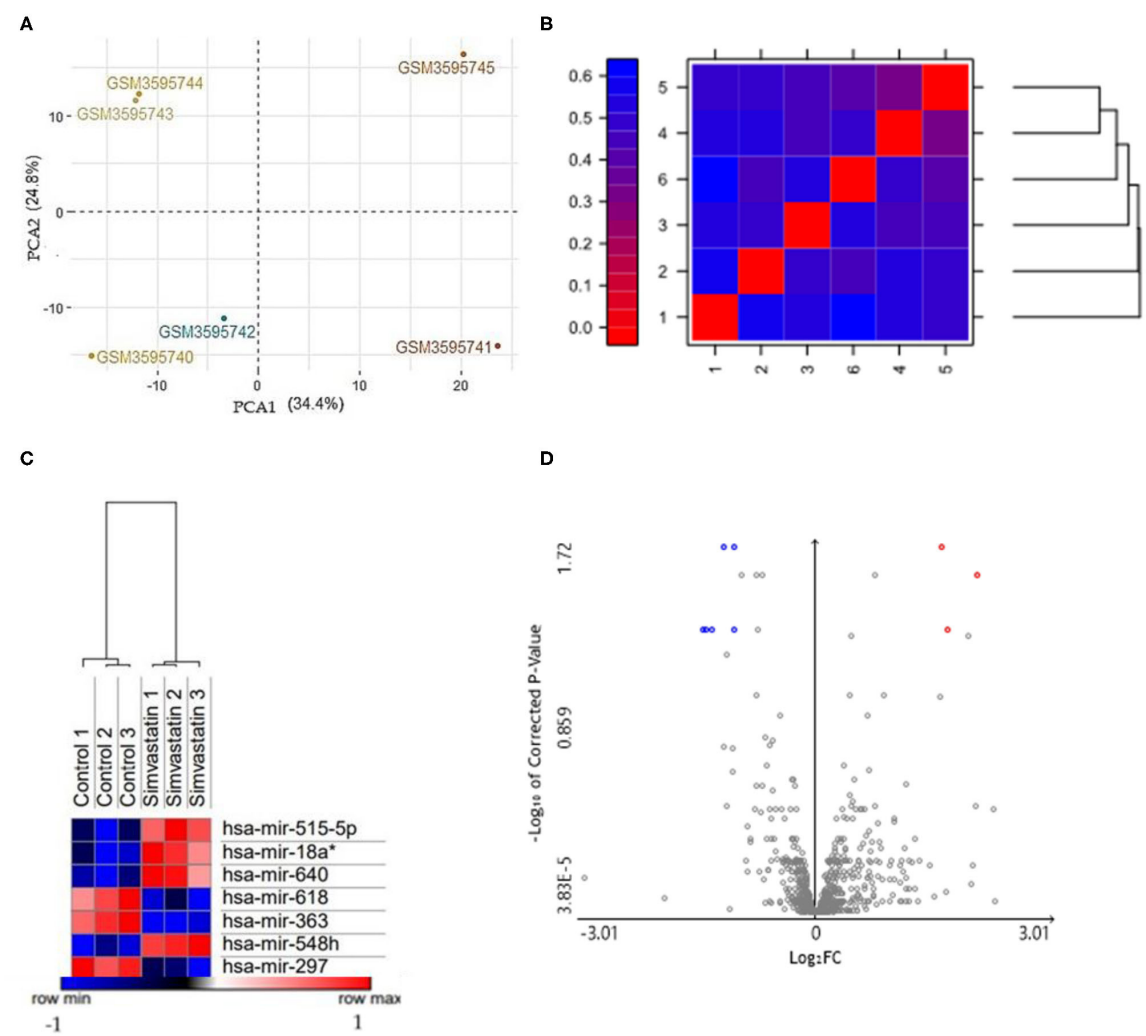
**(A)** PCA based on microarrays showed that samples were grouped. The HUVEC control (GSM3595740; GSM3595741; GSM3595742) and HUVEC cells treated with 10μM simvastatin (GSM3595743; GSM3595744; GSM3595745) were separated by PC2. **(B)** False color heat maps indicating distances between matrices. 1 (Control 1); 2 (Control 2); 3 (Control 3); 4 (Simvastatin 1); 5 (Simvastatin 2); 6 (Simvastatin 3). **(C)** Hierarchical clustering heat maps with differentially expressed miRNAs in both conditions: control and simvastatin treated. In the graph, the red samples represent the highly expressed values while the blue ones show the low expressed values. The values are expressed in Log2. **(D)** Volcano plot of the differentially expressed miRNAs. The gray points represent genes with no significant difference. The red points represent upregulated genes screened based on the fold change > 1.0 and a corrected *p*-value of < 0.05. The green points represent downregulated genes screened based on the fold change > 1.0 and a corrected *p*-value of < 0.05.

To determine the quality control of our analysis, two PCA plots and a false-color heat map were generated. PCA shows the separation of the groups in the two conditions evaluated, simvastatin-treated HUVEC cells, and control (untreated) HUVEC cells (Figure 1A). Along with this, the false heat map indicates the distances between the matrices, calculated between the mean distances between the data (Figure 1B). Subsequently, the differential expression of miRNAs from the GSM126290 database is shown. The criteria used were *p* < 0.05 and logFC > 1 (Table 1). A total of seven miRNAs were identified as differentially expressed, three miRNAs upregulated and four miRNAs downregulated, between the conditions: 10-μM simvastatin-treated and control cells. This was represented in a hierarchical clustering heat map (Figure 1C). Along with this, the volcano plot showing the dataset and the differentially expressed samples is shown (Figure 1D).

**Table 1:** Differentially expressed miRNAs in HUVEC cells treated with 10μM simvastatin for 24 h.

**miRNA_ID**	**logFC**	**AveExpr**	**T**	**p value**	**adj. p val**	**B**	**Expression**
hsa-miR-515-5p	−1.07	10.1	−6.94	0.000662	0.0471	0.255	Downregulated
hsa-miR-18a^*^	−1.43	9.06	−7.18	0.000559	0.0471	0.43	Downregulated
hsa-miR-640	−1.35	9.31	−7.06	0.00061	0.0471	0.34	Downregulated
hsa-miR-618	1.72	5.82	6.81	0.00073	0.0471	0.155	Upregulated
hsa-miR-363	1.64	10.7	13.4	2.2E-05	0.0191	3.48	Upregulated
hsa-miR-548h	−1.19	12.9	−10.9	6.43E-05	0.0191	2.55	Downregulated
hsa-miR-297	2.1	11.7	8.53	0.000233	0.026	1.32	Upregulated

*logFC, log2 fold change; AveExpr, average log2 expression; T, moderated t-statistic; p-value, raw p-value; adj. p-value, adjusted p-value; B, log-odds*.

### Functional and Pathway Enrichment Analysis of Differentially Expressed miRNAs

**Figure 2:**
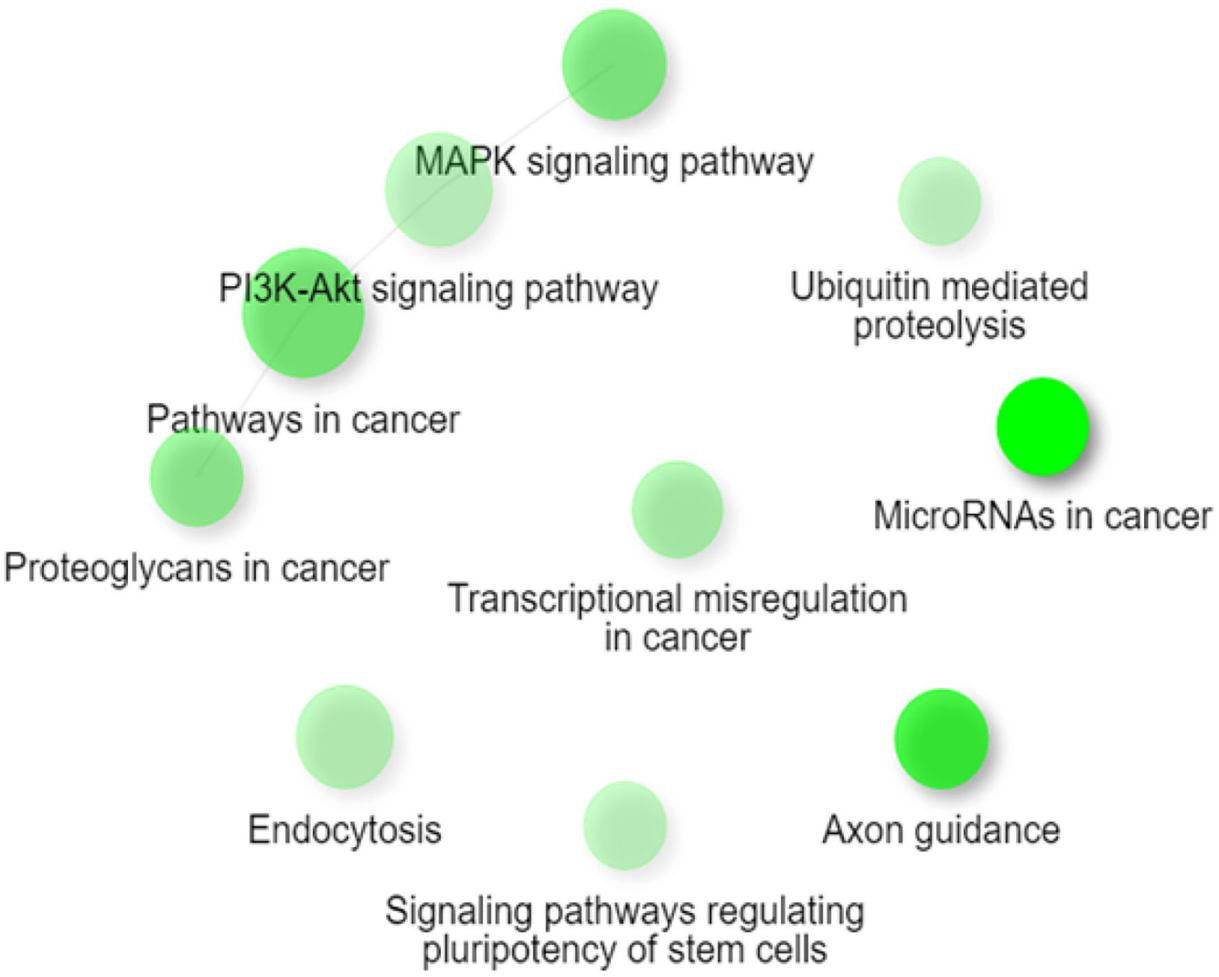
Network figure showing the relationship between the enriched pathways obtained from the classification from KEGG. The nodes in green correspond to an enriched pathway, and their size is related to a set of larger genes. MAPK signaling, PI3K-Akt signaling pathway, pathways in cancer, and proteoglycans in cancer are connected as they share ≥ 20% of genes. Figure extracted from ShinyGO v0.61.

Gene Ontology and Pathway Enrichment analysis were made to explore the biological pathways in which deregulated miRNAs would be implicated. The first step was to determine the target genes for differentially expressed miRNAs, using the mirDIP 4.1 program. This program brings together 30 different resources. A total of 4,756 target genes were found with extremely high score. Later, the 4,756 genes were used for ontological genes and enrichment pathways. Gene Ontology enrichment analysis classified them into three groups, a biological process group, a cellular component group, and a molecular function group, which are shown in Table 2. The biological processes that showed the highest number of genes were regulation of biosynthetic processes (1,055 genes), regulation of macromolecule biosynthetic processes (1,010 genes), and positive regulation of nitrogen compound metabolic processes (819 genes). The cellular components genes are mostly associated with nuclear part (1,060 genes), nucleoplasm (869 genes), and nuclear lumen (976 genes). The most presented molecular functions were transcription regulator activity (543 genes), regulatory region nucleic acid binding (301 genes), and transcription regulatory region DNA binding (299 genes). Pathway enrichment analysis shows miRNAs in cancer, axon guidance, and pathways in cancer, in higher probability, which are shown in Table 3 and Figure 2.

**Table 2:** Gene ontology enrichment analysis of differentially expressed miRNAs was divided into three groups: biological processes, cellular components, and molecular functions.

**Functional category**	**E. FDR**	**N. G. S**	**N. G. T**
**BIOLOGICAL PROCESS**			
Nervous system development	4.6E-72	696	2,474
Positive regulation of metabolic process	2.4E-60	911	3,789
Positive regulation of macromolecule metabolic process	2.4E-60	858	3,498
Regulation of biosynthetic process	2.2E-56	1,055	4,687
Regulation of macromolecule biosynthetic process	2.2E-56	1,010	4,426
Positive regulation of nitrogen compound metabolic process	2.2E-56	819	3,351
Regulation of cellular biosynthetic process	6.4E-56	1,040	4,610
Neurogenesis	7.9E-56	495	1,683
Cell development	1.8E-55	604	2,230
Regulation of nucleobase-containing compound metabolic process	6.7E-55	1,014	4,482
**CELLULAR COMPONENT**			
Nuclear part	1.9E-44	1,060	4,966
Nucleoplasm	3.1E-44	869	3,861
Nuclear lumen	1.7E-41	976	4,545
Neuron part	2.1E-27	440	1,808
Synapse	5.5E-24	326	1,268
Axon	7.7E-21	190	639
Neuron projection	3.5E-20	333	1,371
Plasma membrane bounded cell projection	5.8E-19	481	2,214
Cell projection	8.0E-19	493	2,287
Synapse part	1.5E-18	257	1,004
**MOLECULAR FUNCTIONS**			
Transcription regulator activity	7.5E-37	543	2,183
Regulatory region nucleic acid binding	3.1E-34	301	1,002
Transcription regulatory region DNA binding	9.6E-34	299	1,000
Sequence-specific DNA binding	1.1E-33	338	1,189
RNA polymerase II regulatory region DNA binding	1.3E-33	260	823
DNA-binding transcription factor activity	4.9E-33	455	1,793
RNA polymerase II regulatory region sequence-specific DNA binding	1.2E-32	256	816
Sequence-specific double-stranded DNA binding	1.2E-31	276	920
Transcription regulatory region sequence-specific DNA binding	1.4E-31	266	875
DNA-binding transcription factor activity, RNA polymerase II-specific	1.4E-31	427	1,673

*E. FDR, enrichment FDR; N. G. S, number of genes selected; N. G. T, number of genes total*.

**Table 3:** Pathway enrichment analysis of differentially expressed miRNAs.

**Functional category**	**E. FDR**	**N. G. S**	**N. T. G**.
**ENRICHMENT PATHWAYS**			
MicroRNAs in cancer	5.5E-13	62	150
Axon guidance	1.5E-10	65	181
Pathways in cancer	5.4E-09	134	528
MAPK signaling pathway	8.4E-09	86	295
Proteoglycans in cancer	1.6E-08	64	198
Transcriptional misregulation in cancer	5.2E-08	60	186
Endocytosis	8.6E-08	72	244
PI3K-Akt signaling pathway	1.1E-07	94	353
Ubiquitin-mediated proteolysis	1.3E-07	47	135
Signaling pathways regulating pluripotency	1.3E-07	48	139
of stem cells			

*E. FDR, enrichment FDR; N. G. S, number of genes selected; N. G. T, number of genes total*.

### miRNA Target Prediction for ROCK1 and ROCK2 Kinases

**Figure 3:**
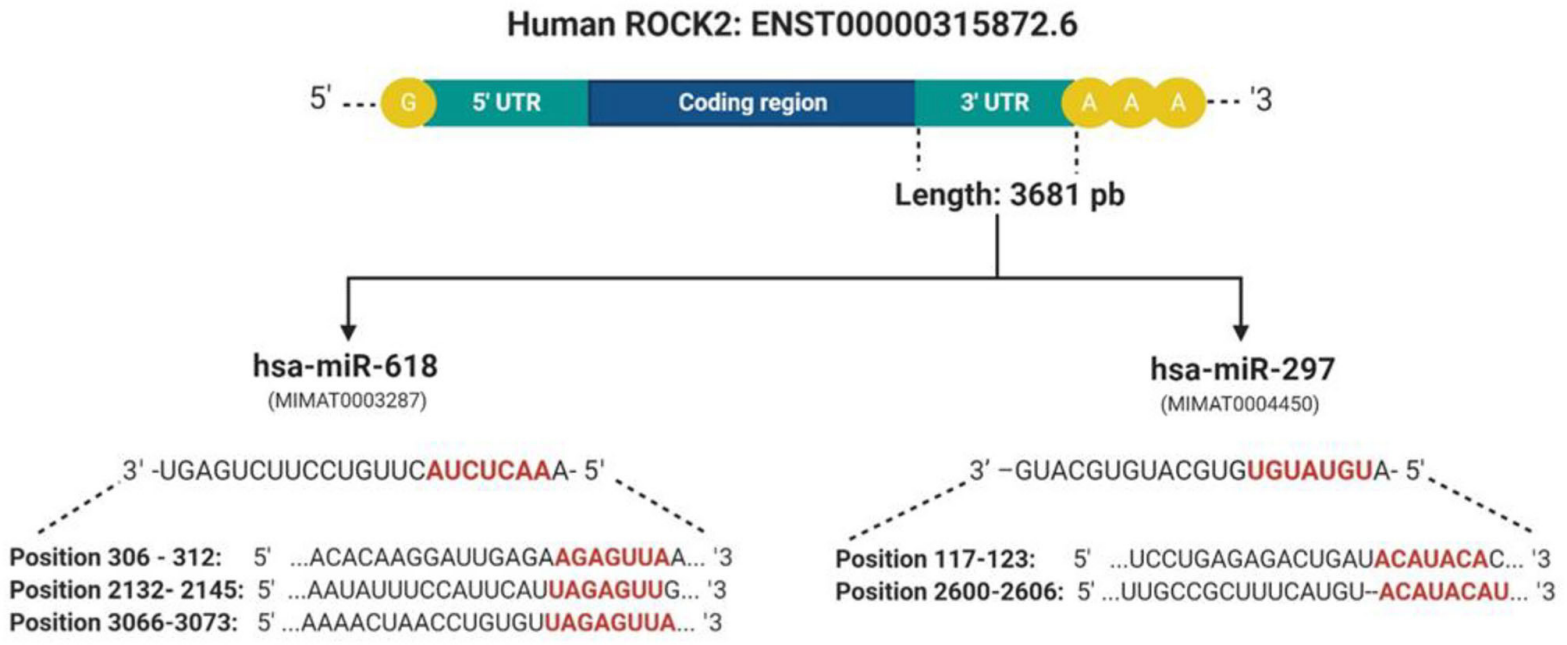
Presentation of complementary sites and base pairs of the miRNAs hsa-miR-618 and hsa-miR-279 with the 3′UTR sequence of the ROCK2 kinase. These were predicted by software: TargetScan, miRDB, and mirDIP 4.1. Created with BioRender.com.

To determine the interaction between the upregulated miRNAs hsa-miR-618, hsa-miR-363, and hsa-miR-297, with the 3′UTR sequence of ROCK1 and ROCK2 kinases, three online platforms were combined, TargetScan, miRDB, and mirDIP 4.1, considering the most important metrics of each program (Figure 3 and Table 4). The strategy was to specifically target ROCK1 and ROCK2 kinases, due to the major role these proteins play in atherosclerotic pathology and also in the pleiotropic effects exerted by statins. However, genes associated with cholesterol metabolism (*ABCA1, APOE, LDLR, SCAP, SREP2, PCSK9*, and *VLDLR*), as well as genes related to eNOS pathways (*ROCK2, PI3K*, and *AKT*), were also evaluated. These genes are not presented as predicted targets of miRNAs.

**Table 4:** Software and metrics used to determine the binding of miRNAs with ROCK2.

**miRNAs**	**Fold change**		**Gene target**	**TargetScan**		**miRDB**		**mirDIP 4.1**
	**LogFC**	**Expression**		**S. T**	**C++S**	**S. L**	**T. S**	
hsa-miR-618	1.75	Up	ROCK2	7mer-A1	−0.15	306	83	X
				7mer-m8	−0.04	2,139		
				8mer	−0.01	3,066		
hsa-miR-297	2.1	Up	ROCK2	7mer-m8	−0.06	117	88	X
				7mer-m8	−0.01	2,600		

*S.T, site type; C++S, context score; S.L, seed location; T.S, target score*.

## Discussion

In the present *in-silico* study, we analyzed data collected from the GEO database (GSE126290) in which the differential expression of miRNAs between two conditions was evaluated: HUVEC cells treated with simvastatin at 10 μM and control cells (without treatment) for 24 h. The bioinformatic analysis on differential expression analysis of miRNAs showed the deregulation of a group of seven miRNAs (hsa-miR-515-5p, hsa-miR-18a^*^, hsa-miR-640, hsa-miR-618, hsa-miR-363, hsa-miR-548h, and hsa-miR-297) considering as cutoff criteria a logFC > 1 and FDR *p*-value < 0.05. Among the group of seven miRNAs, four are downregulated (hsa-miR-515-5p, hsa-miR-18a^*^, hsa-miR-640, and hsa-miR-548h) and three are upregulated (hsa-miR-618, hsa-miR-297, and hsa-miR-363). Liang et al. (38) performed a differential expression analysis from the same data set presented in this paper. Their results showed that miRNA hsa-miR-652-3p inhibits ISL1, a protein involved in the decrease of NO bioavailability in endothelial cells. However, this miRNA is not upregulated in our analysis. One of these differences is due to their cutoff values considered: *p*-value of 0.1 and fold change ± 0.2. This would also establish a difference in the amount of deregulated miRNAs, since they present a total of 167, in relation to our analysis which were 7.

Our work supports previous reports on the effect of statins on the dysregulation of miRNA expression and also proposes the way in which statins can exert pleiotropic effects associated with the RhoA/ROCK kinase pathway. These miRNAs are modulating various processes associated with CVDs (39, 40). We observed that simvastatin can modulate seven miRNAs in endothelial cells, and two that are upregulated could modify their expression through binding to the 3′UTR region of the ROCK2 kinases. Nowadays, there are several tools available to predict miRNA targets based mainly on detecting the complementarity of the miRNA sequence with the 3′UTR region of the target gene (41). However, many of these predictions show false negatives in the experimental validation. One strategy to maximize performance and minimize false results *in vitro* and *in vivo* functional experiments is the union of bioinformatics tools for miRNA-target prediction that will allow increasing the specificity and precision of the analysis (42). For this reason, three prediction programs were used together: TargetScan, miRDB, and mirDIP 4.1. All three tools showed that hsa-miR-618 and hsa-mir-297 had ROCK2 as a target. The hsa-miR-618 miRNA shows complementarity in three regions of the 3′UTR of ROCK2. Studies have shown that the more seed bonds it has in the 3′UTR, the stronger the miRNA-induced regulation (43). On average, the change in Log2 was linearly correlated with the number of seed regions, suggesting that the effect of seeds is independent and multiplicative (44). For this reason, we can suggest that hsa-miR-618 would have a greater effect on the 3′UTR.

Considering that many of the pathologies associated with the vasculature are related to morphological changes that can be regulated by the interaction of hemodynamic forces, one of the limitations of our work is that the database used contains data from an *in vitro* model and not from an *in vivo* model. Also, in the vasculature there is presence of different cell types that contribute to the physiopathology. An example of this, in the adhesion of leukocytes, is the production of inflammatory cytokines by macrophages, etc. However, many authors agree that HUVEC is a model of representative study of the vasculature and also it has allowed the study of physiological and pathological effects in isolation as in coculture with leukocytes and smooth-muscle cells (45, 46). For this reason, it is important to consider validating these results *in vitro* and *in vivo* models.

ROCK2 is a 160-kDa serine/threonine kinase protein, and its functions are the regulation of smooth-muscle contraction, organization of the actin cytoskeleton and stress fibers, formation of focal adhesions, and retraction of neurons and cellular adhesions (47). Therefore, its function in different cells and how it contributes to different pathologies through mechanisms mediated by miRNAs have been studied. Liu et al. (48) demonstrated that hsa-miR-122 can inhibit the proliferation of prostate carcinoma cells due to the negative regulation of ROCK2 expression. Furthermore, the hsa-mir-185/ROCK2 pathway was shown to have potential to improve therapies in hepatocellular carcinoma, through metastasis inhibition (49). However, miRNAs hsa-miR-618 and hsa-miR-297 have not been experimentally validated as ROCK2 targets. The hsa-miR-618 has been reported to be unregulated in pathologies as epilepsy (50) and squamous cells from head and neck carcinomas (51). The case of hsa-miR-297 has been associated with prostate cancer (52) and with colorectal carcinoma, specifically with drug resistance (53).

The set of differentially expressed miRNAs and their gene ontological analysis showed the following biological processes: regulation of biosynthesis processes, regulation of biosynthetic macromolecule processes, and positive regulation of nitrogen compound metabolic processes; this last process could be related to the gene's enrichment involved in the mechanisms associated with the ability of statins to increase the bioavailability of NO, through the inhibition of ROCKs (9). The increased expression of ROCKs reduces the expression of eNOS, and the inhibitors (Y-27632 and fasudil) have been shown to increase eNOS (54). In the case of the statins, the increased expression of eNOS was not due to FPP and LDL-C; it was due to inhibition of GGP involved with RhoA and ROCK signaling (6).

Other biological processes and cellular components highlighted in the analysis are neurogenesis, development of the nervous system, and cellular development. Several studies in animal models of spontaneous intracerebral hemorrhage that have been treated with statins have improved neurological function, reduced cerebral edema effects, increasing angiogenesis, and neurogenesis, and have decreased the infiltration of inflammatory cells. This supports the possible neuroprotective effects of statins (55–57).

Statins may also exert their pleiotropic effects through Kruppel-like factor-2 (KLF2). Statins induced the increase in mRNA expression of KLF2 in endothelial cells, which is necessary for eNOS expression (58). This could be associated with the results of molecular functions obtained like the transcription regulator activity in ontological gene analysis.

Inside the analysis associated with the biological pathways, the following ones might be highlighted: signaling pathways regulating pluripotency of stem cells, axon guidance, PI3K-AKT signaling pathways, and miRNAs in cancer. The signaling pathways regulating pluripotency of stem cells has been associated with the transforming growth factor (TGF-B) superfamily, performing important functions during the differentiation of vascular progenitor cells derived from mouse embryonic stem cells (59). Statins can exert pleiotropic effects by increasing the mobilization of endothelial progenitor cells. Damaged endothelial progenitor cells are associated with impaired endothelial function and reduced NO levels. Studies show that prehypertension and hypertension in patients are related to early senescence of progenitor cells and impaired endothelial function (60). Another route mentioned is the axon guidance, which could represent a key stage in the formation of the neural network. This mechanism has been associated with the Rho GTPase pathway through the reorganization of the cytoskeleton that determines the direction that will guide the growth cone (61). A study conducted on brain slices of oxygen-glucose-deprived cells that were exposed to fasudil (ROCK inhibitor) showed an improvement in neuronal viability (62, 63). These findings could be linked to the neuroprotective effects that statins would have on the neurovascular system. Furthermore, the PI3K-AKT signaling pathway corresponds to one of those affected by ROCK inhibition by statins as mentioned above. ROCK is a negative regulator of AKT, possibly through activation of phosphatase and the homolog of tensin (64). Also, the regulation of AKT is known to influence the expression of eNOS and thus an increase in the bioavailability of NO. This effect has been shown to contribute to a decrease in the proliferation of smooth-muscle cells, a key mechanism in the development of atherosclerosis (65). Studies have revealed that statins modulate miRNAs associated with influence the change from senescent contractile phenotype to proliferative phenotype. This could be associated with the enrichment of the miRNA pathway in cancer (66).

**Figure 4:**
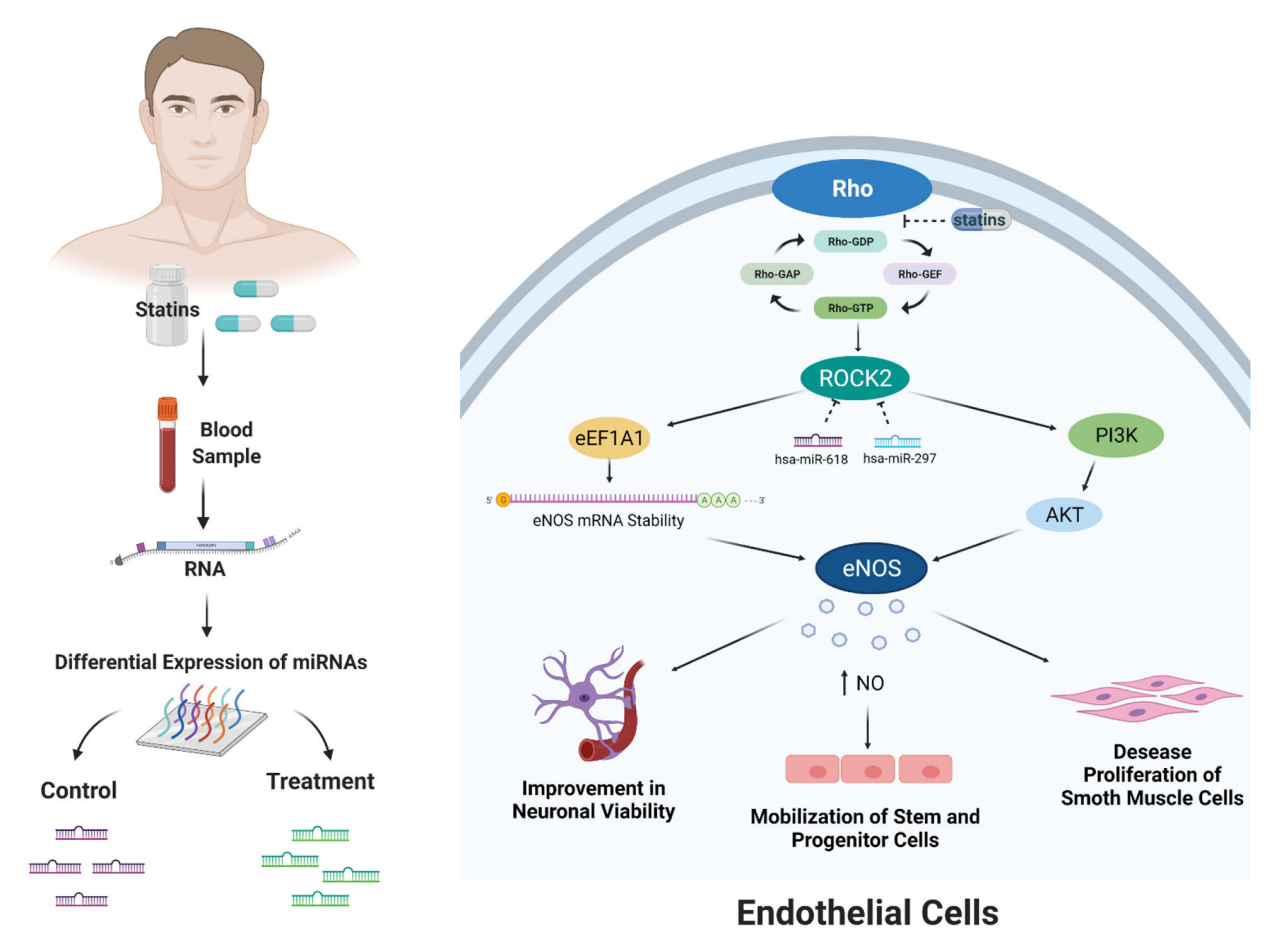
Bioinformatic analysis showed that miRNAs modulated by statin treatment could exert the pleiotropic effects. These effects would occur due to the posttranscriptional inhibition of ROCK2 kinase by the miRNAs hsa-miR-618 and hsa-miR-297. ROCK2 kinase expression has been described by two mechanisms: stability of eNOS messenger RNA and increased activity by activation of phosphatidylinositol 3-kinase (PI3K)/AKT. The increased activity and expression of eNOS produce an increase in the bioavailability of NO. This effect of increasing NO affects improvement in neuronal viability, mobilization of stem and progenitor cells, and decreased proliferation of smooth muscle cells. Created with BioRender.com.

Finally, *in-silico* analysis has shown that statins might exert pleiotropic effects through the modulation of miRNAs that would inhibit ROCK2 kinase and also generate atheroprotective effects in endothelial cells (Figure 4). Although our work proposes new miRNAs that regulate the RhoA/Rock kinase pathway through an *in-silico* approach, it is necessary to continue with the experimental validation of the miRNAs expressed differentially in endothelial cells through *in vitro* and *in vivo* study models. In addition, the functional role of hsa-miR-618 and hsa-miR-297 miRNAs as inhibitors of ROCK2 gene expression and in the mediation of pleiotropic effects should be evaluated.

## Conclusion

In this work, we report that simvastatin treatment deregulates the expression of seven miRNAs (hsa-miR-515-5p, hsa-miR-18a^*^, hsa-miR-640, hsa-miR-618, hsa-miR-363, hsa-miR-548h, and hsa-miR-297) in HUVEC cell culture. *In-silico* analysis shows that the miRNAs hsa-miR-618 and hsa-miR-297 are upregulated, and the 3′UTR region of ROCK2, an important protein of the RhoA/Rock kinase pathway involved in modulation of pleiotropic effects exerted by statins, is predicted as a potential target for both miRNAs. In addition, functional analysis and enriched pathways revealed an important association with the pleiotropic effects produced by statins and with the inhibition of RhoA/ROCKs. These results open a new way to understand how statins, through the deregulation of the miRNA expression, might cause an atheroprotective effect through the inhibition of ROCK2 kinase.

In summary, our work through the use of bioinformatics tools contributes with new potential candidates that could regulate the pleiotropic effects in response to statin treatment. However, the functional role of hsa-miR-618 and hsa-miR-297 miRNAs and their mediation of pleiotropic effects must be evaluated by validating *in vitro* and *in vivo* models.

## Data Availability Statement

Publicly available datasets were analyzed in this study. This data can be found here: Accession number GSE126290.

## Author Contributions

KL and CR performed the bioinformatics analysis. KL wrote the manuscript. KS and LS contributed in writing—review and editing. All authors read and approved the final manuscript.

## Conflict of Interest

The authors declare that the research was conducted in the absence of any commercial or financial relationships that could be construed as a potential conflict of interest.

## Publisher's Note

All claims expressed in this article are solely those of the authors and do not necessarily represent those of their affiliated organizations, or those of the publisher, the editors and the reviewers. Any product that may be evaluated in this article, or claim that may be made by its manufacturer, is not guaranteed or endorsed by the publisher.
